# Thoracic Endovascular Aneurysm Repair and Tracheal Stenting for Respiratory Failure due to a Thoracic Aortic Aneurysm: A Case Report

**DOI:** 10.1177/15385744221085814

**Published:** 2022-04-01

**Authors:** Nadia A.G. Hakkenbrak, Maarten Truijers, Irene Thomassen

**Affiliations:** 1 Department of vascular surgery, Amsterdam UMC, 1209 Location VU Medical Centre, Amsterdam, The Netherlands; 2 Department of vascular surgery, 1140 Northwest Clinics, Alkmaar, The Netherlands; 3 Department of vascular surgery, 3670 Spaarne Gasthuis, Haarlem, The Netherlands

**Keywords:** tracheal compression, aortic aneurysm, respiratory insufficiency

## Abstract

An 82-year-old male was referred to the emergency department for severe respiratory distress. Computed tomography angiography showed tracheal compression due to a large ruptured saccular aneurysm of the descending thoracic aorta. Emergency Thoracic Endovascular Aneurysm Repair (TEVAR) was performed. To reduce tracheal compression, an endotracheal stent was placed (silicone Dumon^©^). Following surgery, respiratory function improved. Two days after the surgery, the patient refused further invasive treatment, including mechanical mucus aspiration from the endotracheal stent, and palliative sedation was initiated. Conventional treatment to reduce tracheal compression by a saccular aortic aneurysm is open surgical aneurysm repair. If open repair is contraindicated because of patient age, comorbidity, or in case of severe hemodynamic instability following aneurysm rupture, TEVAR with endotracheal stent placement may serve as a bridge to definite surgery to reduce tracheal compression.

## Introduction

Mycotic aortic aneurysms are a rare presentation of aortic disease, taking account of less than 1% of all aneurysms in the Western world.^1,2^ Conventional treatment consists of open aneurysm repair. Thoracic Endovascular Aneurysm Repair (TEVAR) is considered a valuable alternative in patients unfit for open repair or as bridge to definitive surgery.^2,3^ In this case report, we describe a rare case of respiratory failure as the result of tracheal compression due to a saccular, mycotic thoracic aortic aneurysm.

## Case description

An 82-year-old male presented with fever and joint pain. Blood cultures showed a *Staphylococcus aureus* bacteremia, and intravenous flucloxacillin was prescribed. Previous medical history revealed an ischemic cerebral vascular accident, psoriasis vulgaris, and corneal transplantation for which the patient used acetylsalicylic acid, dipyridamole, and valacyclovir.

**Figure 1.**
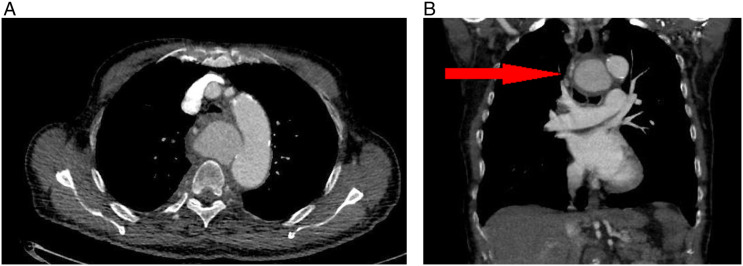
Thoracic aortic aneurysm with pending rupture (A), causing compression of the trachea (arrow) (B).

After 2 weeks of antibiotic treatment, the patient experienced progressive respiratory distress. Laboratory testing displayed an elevated C-reactive protein (180 mg/l) and leukocytosis (13 × 10^9^/l). Chest X-ray revealed a widened mediastinum, without signs of infection. An additional computed tomography angiography scan (CTA scan) showed a ruptured mycotic saccular aneurysm of the descending thoracic aorta, diameter of 55 mm, originating 4 cm distal to the left subclavian artery, causing compression of the trachea and esophagus (Figure 1).

**Figure 2.**
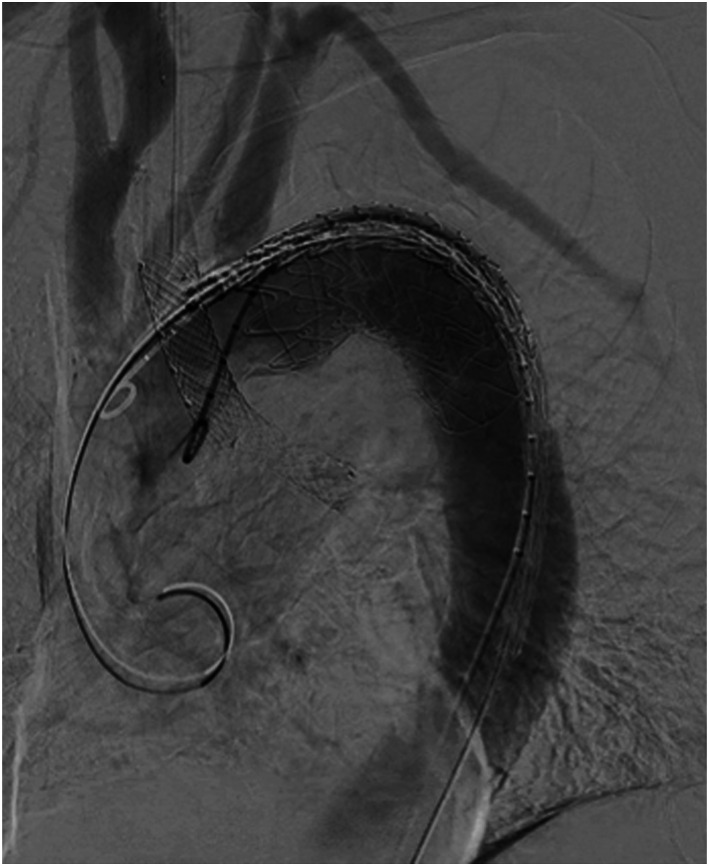
Completion angiography after TEVAR.

Emergency Thoracic Endovascular Aortic Repair (TEVAR) procedure was performed (Gore^©^ CTAG^©^ 37 mm x 10 cm) just distal from the left subclavian artery. Completion angiography showed exclusion of the aneurysm without signs of endoleak (Figure 2).

**Figure 3.**
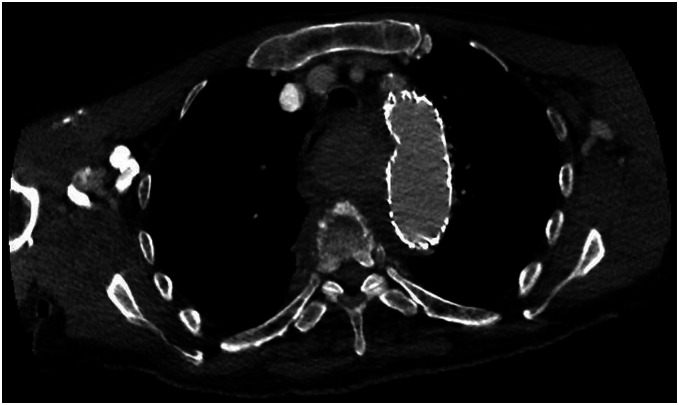
CTA scan showing progressive compression of the trachea and esophagus, stable aneurysm diameter (56 mm) and suspicion of a type 1b endoleak.

Several hours following the procedure, the patient deteriorated, suffering from tachycardia, tachypnea, and respiratory failure. Imaging (CTA scan) showed consistent compression of the trachea and esophagus (Figure 3). Emergency bronchoscopy showed severe obstruction of the distal tracheal lumen, carina, and proximal left and right bronchus. To dilate the tracheal lumen, a silicone Dumon^©^ stent (18 × 14 × 14 mm) and additional fully covered expandable Microtech^©^ nitinol Y stent (16 × 13 × 13 mm) were deployed in the trachea. Following tracheal stent placement, respiratory function improved drastically and the patient was readily detubated. Hereafter, frequent mechanical aspiration was needed to prevent tracheal stent occlusion as a result of mucus accumulation. Thoracostomy to reduce compression by the hematoma was considered to allow tracheal stent removal and restore cough reflex and cilia function. However, because of the prolonged and long anticipated hospitalization, the patient refused further invasive treatment and deceased following palliative sedation.

## Discussion

Mycotic aortic aneurysms (MAAs) most often develop due to bacteremia in patients with pre-existing atherosclerotic aortic aneurysms.^1^ Seeding of disrupted aortic intima or mural thrombus, infection in related organs, penetrating trauma, or bacterial endocarditis, may cause an aggressive local infection with rapid expansion of the arterial wall.^1^ Symptoms are often nonspecific such as fever, sepsis, and less frequently chest or shoulder pain.^4^ On CTA scan, a saccular aneurysm with an irregular contour in absence of extensive atherosclerosis or periaortic findings such as soft tissue inflammation is suggestive of a mycotic aneurysm.^5^

Historically, surgical debridement, intravenous antibiotic treatment, and open aorta repair are considered the treatment of choice for MAA. However, recent studies, that is, a European multicentre study on mycotic aortic aneurysms showed durable outcome of endovascular treatment with 91% survival at 30 days, 19% fatal infection-related complications, and 55% 5-year survival rate.^6^ Endovascular repair should be considered a bridge to definite surgical repair in ruptured aneurysms, fever, tracheal- or esophageal fistula, or bleeding.^6^

Tracheal compression due to vascular pathology is very rare and can be congenital, for example, double aortic arch, aberrant subclavian artery, or Kommerell’s diverticulum, or acquired, for example, aneurysm or dissection of the aortic arch or descending thoracic aorta.^7,8^ Literature regarding the treatment of tracheal compression is limited and most often regards case reports describing invasive procedures.^8^ Recent reviews have shown effectivity rates of up to 75% for Dumon^©^ stents treating benign tracheal stenosis.^9,10^ Mucus retention has been described as frequently occurring complication, with a rate up to 24%, and results from mucosa injury after stent placement and tissue hyper granulation.^9,10^ In our case, the patient suffered from severe mucus retention and the inability to clear his airway due to critical illness, advanced age, physical state, and comorbidity. Patient refused further treatment in order to prevent anticipated complications, prolonged hospitalization, and rehabilitation. Hereafter, care was withdrawn. This case report describes tracheal stenting and endovascular treatment as a possible alternative in patients, with tracheal compression due to a ruptured large thoracic MAA, who are unfit for open repair or in acute setting as a bridge to surgery.

## Conclusion

In patients unfit for open surgical repair, tracheal stenting and endovascular treatment is a possible alternative to reduce tracheal compression as a result of a thoracic aortic aneurysm. Mucus accumulation in the endotracheal stent is a known complication following tracheal stent placement and requires frequent mechanical aspiration.
